# New Appliances and Things Medical

**Published:** 1903-08-01

**Authors:** 


### NEW APPLIANCES AND THINGS MEDICAL.

[We shall be glad to receive at our Office, 28 & 29 Southampton Street, Strand, London, W.C., from the manufacturers, specimens of all new preparations and appliances which may be brought out from time to time.]

### KRESOL.

(HAY, STEARN AND CO., KELVINDOCK CHEMICAL WORKS, MARYHILL, GLASGOW.)

THIS powerful antiseptic is a combination of the coal tar derivatives. It has the advantage of not forming a milky solution when mixed with water, and like carbolic acid it mixes in all proportions with water, affording a perfectly clear solution. Two per cent. solutions possess bactericidal qualities which are sufficiently powerful for the ordinary purposes of disinfection. For surgical and domestic purposes Kresol must be regarded as an antiseptic of reliable and powerful properties.

### ENEMA SYRINGE.

(REUTER'S PATENT, 6 WELL STREET, JEWIN STREET, LONDON, E.C.)

REUTER'S Patent Enema Syringe is distinguished from the ordinary types of Higginson's syringe by the introduction of two new features which, from a practical point of view, have much to commend them. The nozzle, which is of vulcanite, delivers a cone-shaped and hollow spray of water, whereby the irrigating stream is evenly and gently distributed over the entire surface of the mucous membrane. In this way the passage can be thoroughly washed out, without fear of damage to its delicate epithelial lining by the forcible injection of water, which is inevitable when the ordinary form of syringe with its powerful jet of water is employed. The second feature of originality in Reuter's patent is the substitution of vulcanite for metal in the construction of the valve. As far as we can see it is practically impossible for this vulcanite valve to get out of order—a virtue which by no means belongs to the metal varieties. The syringe is throughout of first-class material, and we believe that in all respects it will prove a most reliable and useful instrument for hospital and private use.

### SMOKE-CONSUMING KITCHEN RANGE.

(ERNEST E. PITHER AND CO., 36 AND 38 MORTIMER STREET, LONDON, W.)

THE question of soot and smoke in densely populated areas has not hitherto engaged the attention of health-governing bodies as it should have done. Nevertheless, much may be done by private effort, and the introduction of such smoke-consuming fireplaces as those of Messrs. Pither and Co.'s could do much to abate the evil. These ranges have passed the experimental stage, and have been proved to do all they claim. They show that failure of combustion is due, not to want of air, but the absence of a sufficiently elevated temperature, under which condition only the combination of carbon with oxygen can be so complete as to produce the product $CO_2$ alone. No CO gas is found immediately over the fire. Other important recommendations of these ranges are—they burn cheap coal; no heat is wasted; there is constant heat to boiler and oven; they are easy to manage, and no chimney-sweep is required. We can only hope that they will become widely known, for it would seem that such must lead to their wide adoption.

